# Highly functionalized calix[4]arenes *via* multicomponent reactions: synthesis and recognition properties

**DOI:** 10.1039/c9ra03354h

**Published:** 2019-06-24

**Authors:** Reza Zadmard, Ali Akbarzadeh, Mohammad Reza Jalali

**Affiliations:** Department of Organic Chemistry, Chemistry and Chemical Engineering Research Center of Iran P. O. Box 14335-186 Tehran Iran zadmard@ccerci.ac.ir +98 2144787785 +98 2144787719

## Abstract

Multicomponent reactions (MCRs) include several aspects of green chemistry principles, so it is obvious that chemists in different areas are increasingly interested in providing their product by multicomponent approaches. MCRs can be very useful in supramolecular chemistry, especially to produce novel supramolecular derivatives. Therefore, there are several reports of highly-functionalized calix[4]arene derivatives obtained by MCRs instead of conventional stepwise protocols during the last decade. In this paper, we have particularly focused on the exploitation of upper rim and lower rim substituted calix[4]arenes in multicomponent approaches as a facile and convenient synthetic strategy. The value of this method lies in its operational simplicity, mild reaction conditions and structural diversity of the products. Interestingly, in most cases the products afforded by this method offer unique features and applications which are highlighted in the following sections.

## Abstract 2

Multicomponent reactions (MCRs) include several aspects of green chemistry principles, so it is obvious that chemists in different areas are increasingly interested in providing their product by multicomponent approaches.

## Graphical abstract

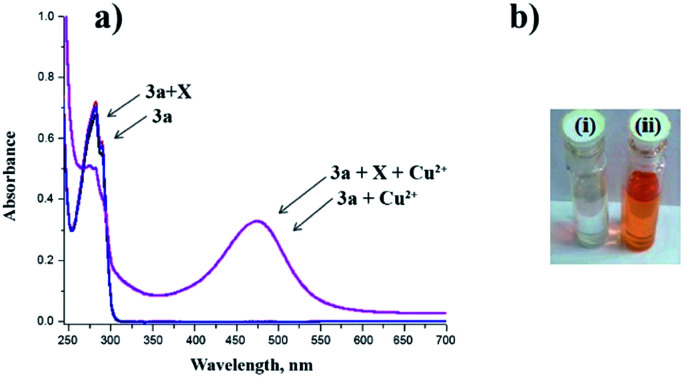

## Introduction

Supramolecular chemistry deals with reversible non-covalent interactions between two or more molecules or ions.^1,2^ It can also be described as chemistry beyond the molecule in view of the fact that simpler chemical species can create more complex structures such as supramolecular entities through the utilization of non-covalent interactions, such as electrostatics, hydrogen binding, van der Waals and donor–acceptor interactions.^3,4^ Both host–guest chemistry and self-assembly are main branches of supramolecular chemistry which are often distinguishable from each other by the size and shape of the species. Generally, the host is the larger molecule which can embrace another one *via* its binding sites whereas the guest is the smaller one embraced by the host. Where there is no significant difference between species in the size and shape, the non-covalent aggregating of two or more species is termed self-assembly.^5^ These types of interactions are the origin of many biological processes and can be used in the development of various synthetic enzymes, drugs and molecular receptors.^6,7^

Calix[4]arenes are basket like cyclic phenol formaldehyde tetramers which create a hollow cavity flanked by a hydrophobic upper rim (aromatic moiety) and a hydrophilic lower rim (hydroxyl groups).^8–10^ The possibility of divers functionalization at lower and/or upper rim as well as unique conformational properties have made calix[4]arenes versatile templates to design outstanding supramolecular structures with several binding sites in an array complementary to a potential guest.^11–13^ The molecular hosts based on calix[4]arene scaffold have been employed in many applications,^14,15^ such as chemical sensing,^16–18^ drug and gen delivery,^19–22^ enzyme mimicking,^23,24^ stationary phase,^25–27^ extractants,^28,29^ enzyme inhibitors,^30–32^ catalysts^33–35^ and *etc.*

Multicomponent reaction (MCR) is defined as a chemical process between more than two substances which afford a more complicated product through formation of multiple bonds in a single step.^36–38^ In the point of view of green organic synthesis, MCRs possess many advantages over conventional approaches such as high atom economy, simple operation, affording highly functionalized molecules in lower reaction and purification steps, saving energy and resources.^39,40^ Nowadays, owing to structural diversity of the multicomponent reaction products, many industrial and academic researchers are interested in developing new biologically active compounds through MCR protocols.^41,42^ A survey of the literature reveals that increasing efforts have been made to develop novel calix[4]arene derivatives through MCRs during the last decade. Also, there are several reports on using calix[4]arene derivatives as organocatalysts in MCRs which are not within the scope of this review.^43–45^ Actually, the purpose of this review is to summarize all of the works conducted using MCR protocols to synthesize novel multi-functionalized calix[4]arene derivatives. As will be shown in further sections, most of the products have special recognition properties.

## Lower rim functionalized calix[4]arene derivatives

**Scheme 1:**
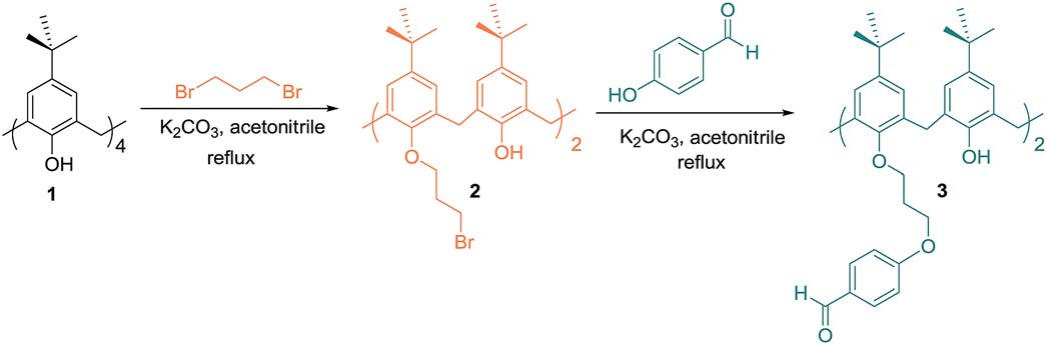
Synthesis of calixarene dialdehyde 3.

As the chemistry of calixarenes is well known nowadays, there are efficient procedures to functionalize both the lower and the upper rim with various functional groups.^46^ In most cases, functional groups have been attached to the lower rim *via* ether linkages. By the introduction of proper bulky groups such as propyl at the lower rim the interconversion among four possible stereoisomers (cone, partial cone, 1,3-alternate and 1,2-alternate) can be prevented.^47^ This way, calix[4]arene structure can be locked in its cone conformation. Compound 3 as a 1,3-dialdehyde *p-tert*-butylcalix[4]arene derivative have been used in several MCRs. In order to achieve compound 3 in excellent yields, *p-tert*-butylcalix[4]arene 1 was refluxed with 5 equivalent of 1,3-dibromopropane in the presence of K_2_CO_3_ in dry acetonitrile for 48–96 h. Then in a similar manner, obtaining 1,3-bis(bromopropane)-*p-tert*-butylcalix[4]arene 2 was refluxed in anhydrous acetonitrile with 4 equivalent of *p*-hydroxybenzaldehyde in the presence of K_2_CO_3_ under a nitrogen atmosphere (Scheme 1).^48^

**Scheme 2:**
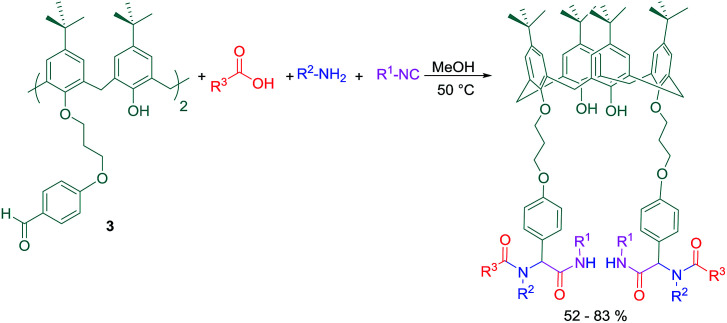
Ugi-4-component reaction of calixarene dialdehyde 3.

The Ugi-4CR is an isocyanide-based multicomponent reaction between an isocyanide, a carbonyl compound, a primary amine, and a carboxylic acid that affords α-acylamido amide derivatives as peptide-like structures. The use of compound 3 in Ugi-4-component reaction (Ugi-4CR) to produce a series of calix[4]arene peptoids was firstly described by Varma and coworkers (Scheme 2).

**Scheme 3:**
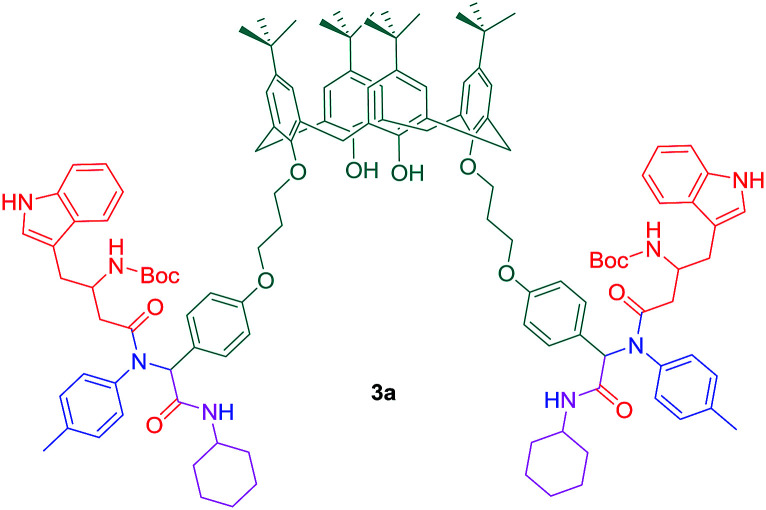
Compound 3a as a selective molecular host for Cu^2+^ cation.

**Fig. 1:**
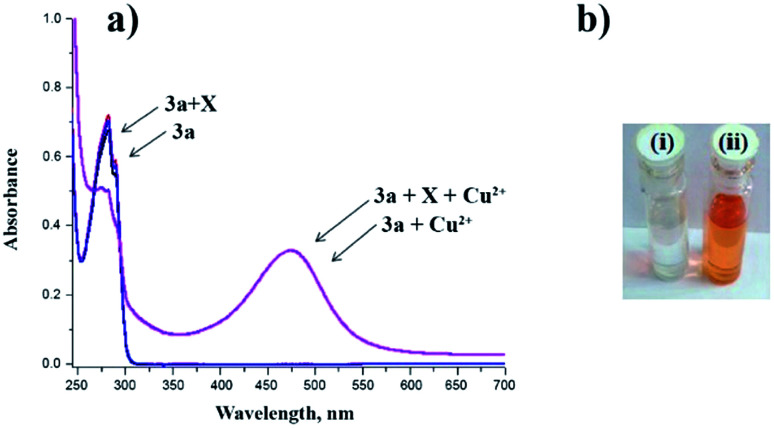
(a) UV-Vis spectra of 3a in the presence of Cu^2+^ and different metal cations (40 equivalent) in CH_3_CN. (b) The color change of the solution, upon Cu^2+^ was added: (i) 3a alone; (ii) 3a + Cu^2+^.

Additionally, the reaction of dialdehyde 3 as the carbonyl compound with some of chiral N-protected alpha-amino acids such as Boc-glycine, Boc-l-alanine, and Boc-l-tryptophan has afforded the products which have the potential to establish one more peptoide bond at the lower rim (Scheme 3). However, the synthesis of similar calix[4]arene derivatives is otherwise very difficult and involves multiple steps. The products obtained by this method have several non-bonding electron pairs which make them able to conduct intermolecular interactions with suitable guests. Among these new calix[4]arene derivatives, compound 3a has shown a significant change in colour and UV absorption toward the Cu^2+^ cation in CH_3_CN, even in the presence of other metal cations (Fig. 1).

The UV-Vis titration revealed an isosbestic point at 296 nm in the range of 0–4 equivalent of Cu^2+^. By Increasing of Cu^2+^ concentration between 4–30 equivalent, a new peak began to appear at 274 nm, as well as the complex band at 474 nm continuously increased and the colour of the solution gradually turned from colourless to orange. Addition of more Cu^2+^ did not lead to any further significant change in the absorbance spectra. The UV-Vis spectral changes of 3a were also observed in the competitive presence of other metal cations which imply that Cu^2+^ : 3a complex is unaffected even in the presence of 40 equivalent of many other cations^49^

**Scheme 4:**
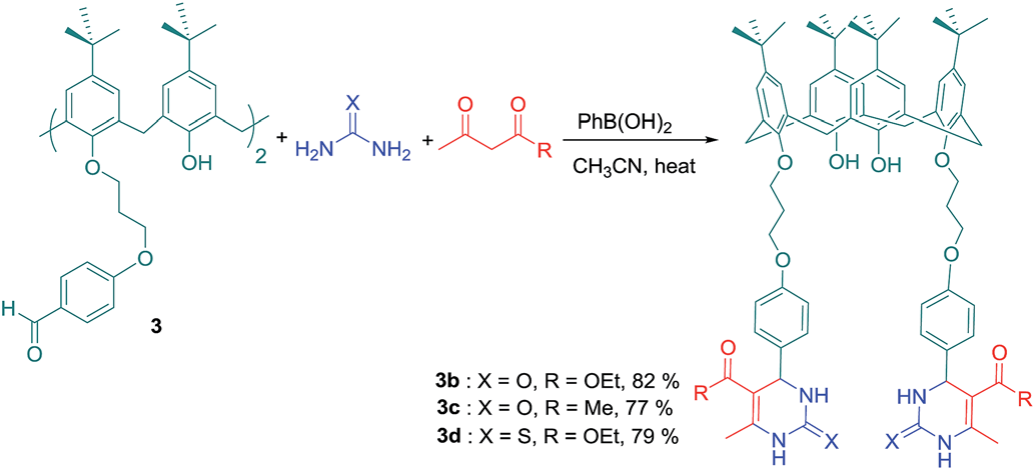
Biginelli-3-component reaction of calixarene dialdehyde 3.

Additionally, Varma and coworkers have successfully used compound 3 to synthesize of dihydropyrimidine derivatives *via* well-known Biginelli-3-Component Reaction (Biginelli-3CR) in good to excellent yields. An extended library of dihydropyrimidine derivatives with important biological properties has been available by Biginelli-3CR which was firstly reported in 1893 by the Italian Chemist Pietro Biginelli.^50^ The reaction of compound 3 with ethyl acetoacetate (or acetyl acetone) and urea (or thiourea) was carried out in acetonitrile as solvent, using phenylboronic acid as a non-toxic and cheap catalyst (Scheme 4). In some cases, significant inter-molecular interactions were revealed by the investigation of aggregation behaviour in non-polar solvents like chloroform.^51^

**Scheme 5:**
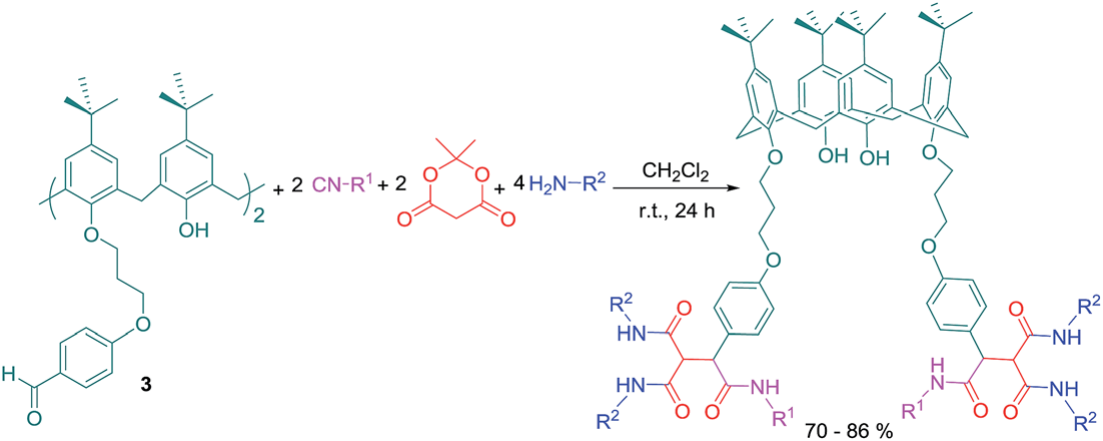
Synthesis of hexaamide derivatives through 4-component reaction of compound 3.

Another reaction was conducted by Zadmard *et al.* between compound 3 with Meldrum's acid, isocyanides and primary amines in dry CH_2_Cl_2_ to produce various calix[4]arene based hexamides in a convenient manner and good yields (Scheme 5).

**Scheme 6:**
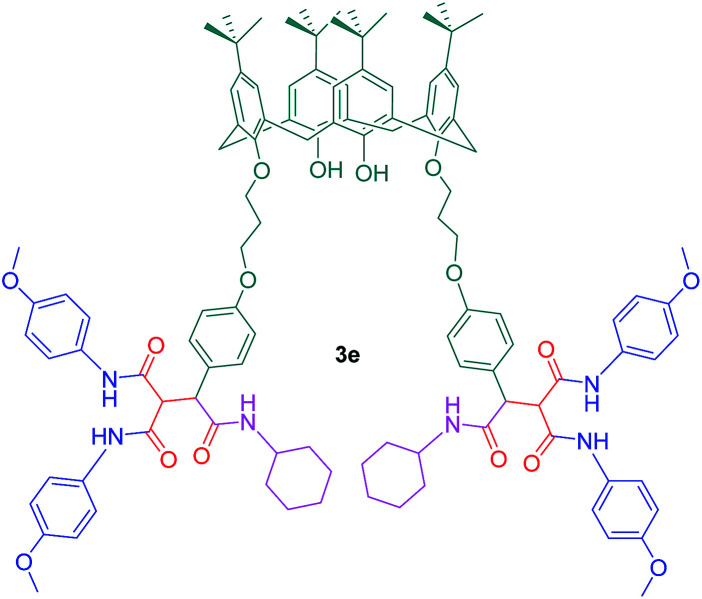
Compound 3e as a BLG host.

**Fig. 2:**
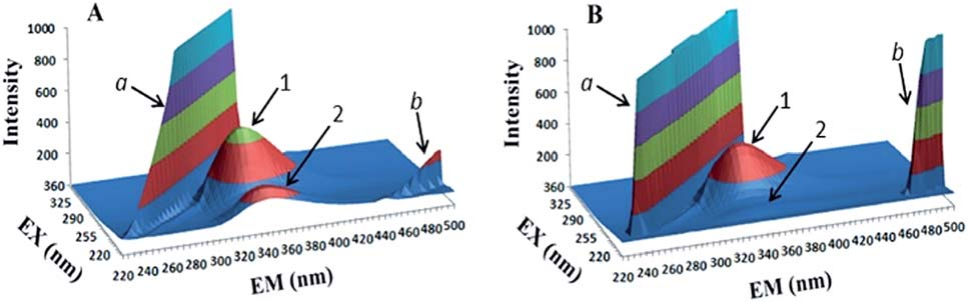
Three-dimensional fluorescence spectra of BLG and 3e : BLG complex.

The presence of amide groups leads to increased hydrogen bonding affinity of the molecule and makes it a suitable host to embrace a guest such as a protein. Moreover, these amide groups can improve the water solubility of hydrophobic calix[4]arene moiety. Fluorescence quenching study revealed that among all the derivatives, compound 3e has the highest binding affinity toward β-lactoglobulin (BLG) protein (Scheme 6). Additionally, three-dimensional (3D) fluorescence spectroscopy investigations proved a complex formation mechanism for the interactions between calix[4]arene hexamides and BLG which did not cause considerable conformational changes in the protein structure (Fig. 2).

3D fluorescence spectroscopy is a suitable method to gather detailed information about the conformational changes in proteins.^52^ Thus, the 3D fluorescence spectra of BLG (A) and 3e : BLG complex (B) are shown in Fig. 2 and the corresponding characteristic parameters are listed in Table 1. In this figure, peaks a and b respectively were assigned as the first-order Rayleigh scattering peak (*λ*_ex_ = *λ*_em_) and second-order Rayleigh scattering peak (2*λ*_ex_ = *λ*_em_). Moreover, peak 1 refers to tryptophan and tyrosine residues in BLG, and peak 2 is the spectral response of the polypeptide backbone structure in BLG protein. Rayleigh scattering peaks a and b were intensified by the addition of compound 3e. This was probably due to formation of 3e : BLG complex which increases the diameter of BLG. Furthermore, the intensity of peaks 1 and 2 significantly decreased without any variation in maximum emission and excitation wavelengths. The obtained results disclosed that any change in microenvironment of the tryptophan and tyrosine did not occur.^53^

**Table 1:** Three-dimensional fluorescence spectral characteristics of BLG and 3e : BLG system

Systems	Peak	Peak position *λ*_ex_/*λ*_em_ [nm nm^−1^]	Intensity
BLG	1	280/340	589
	2	230/340	271
BLG : 3e (1 : 2)	1	280/340	327
	2	230/340	95

**Scheme 7:**
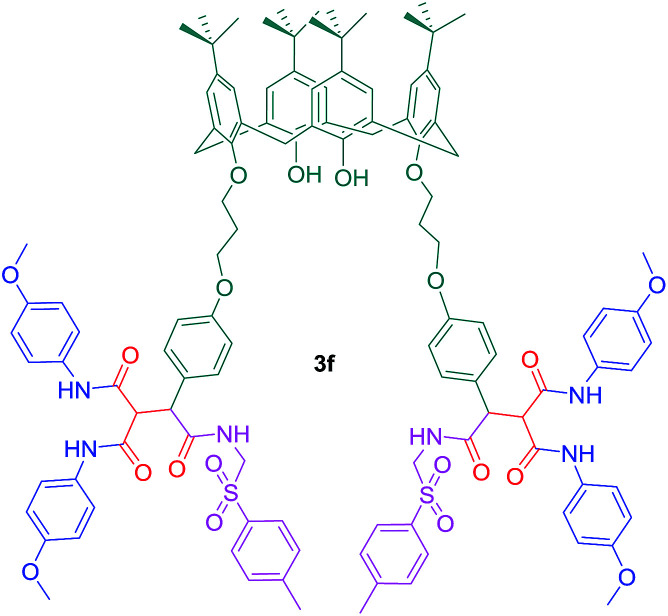
Compound 3f as an inhibitor for Lysozyme enzyme.

Also, the binding affinity of compound 3f toward some proteins has been investigated in a similar study (Scheme 7). Surprisingly, it was found that the binding affinity of 3f toward lysozyme (Lys) is significantly higher than the others (Table 2).

**Table 2:** Binding constants of proteins with compound 3f

Proteins	*K*_A_ (M^−1^)
Lys	2.96 × 10^6^
BLG	1.14 × 10^5^
BSA	8.34 × 10^4^
HSA	7.84 × 10^4^

As most of tryptophan residues in Lys have polar-uncharged and hydrophobic amino acids in their vicinity, it is possible to establish hydrogen bonds and hydrophobic interactions toward compound 3f at the same time. One of the advantages of using compound 3f as a Lys inhibitor is that the formation of Lys : 3f complex cannot be considerably affected by the presence of biologically important metal ions.^54^

**Scheme 8:**
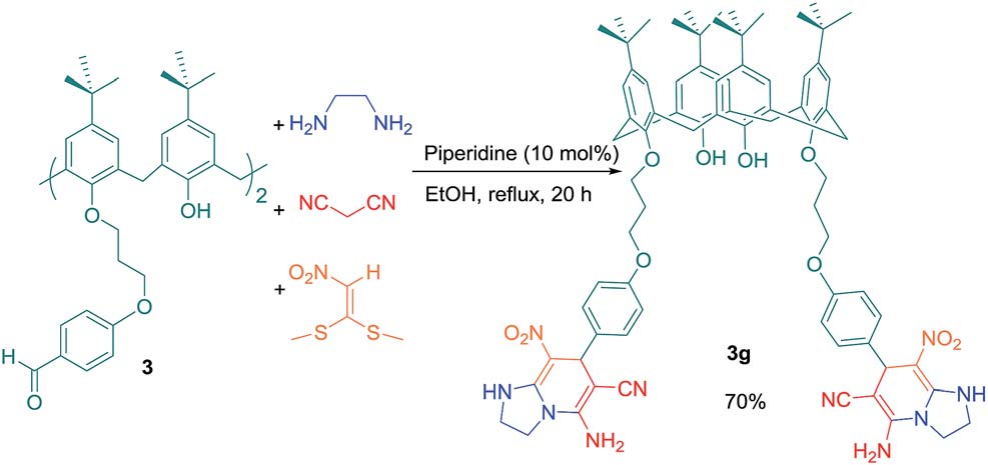
Synthesis of multifunctional calix[4]arene 3g.

In the most recent work, one-pot 4-component reaction of compound 3 with 1,1-bis (methylthio)-2-nitroethylene, 1,2-ethanediamine, and malononitrile was carried out in ethanol as solvent and in the presence of 10 mol% piperidine as a basic catalyst under reflux condition to afford the new multifunctional calix[4]arene 3g (Scheme 8).

**Fig. 3:**
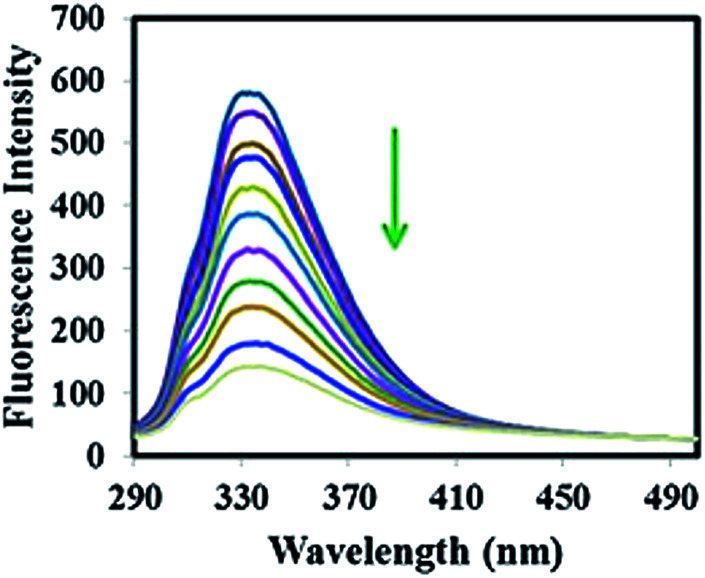
Changes in fluorescence spectra of BLG (1 μM) upon addition of compound 3g (0–3.3 μM).

The binding property of compound 3g with four selected proteins was investigated by fluorescence quenching titration. Among them, BLG showed the most dramatic decrease in the fluorescence intensity without any shift in the maximum emission wavelength (Fig. 3). These results imply a strong interaction between 3g and BLG without considerable conformational changes in the protein structure.

As shown in Table 3, the binding constant for 3g : BLG complex was found to be significantly higher than the binding constant for the three other proteins.^55^

**Table 3:** Binding constants of proteins with compound 3g

Proteins	*K*_A_ (M^−1^)
BLG	1.06 × 10^6^
Lys	2.66 × 10^5^
BSA	9.04 × 10^4^
HSA	5.22 × 10^4^

**Scheme 9:**
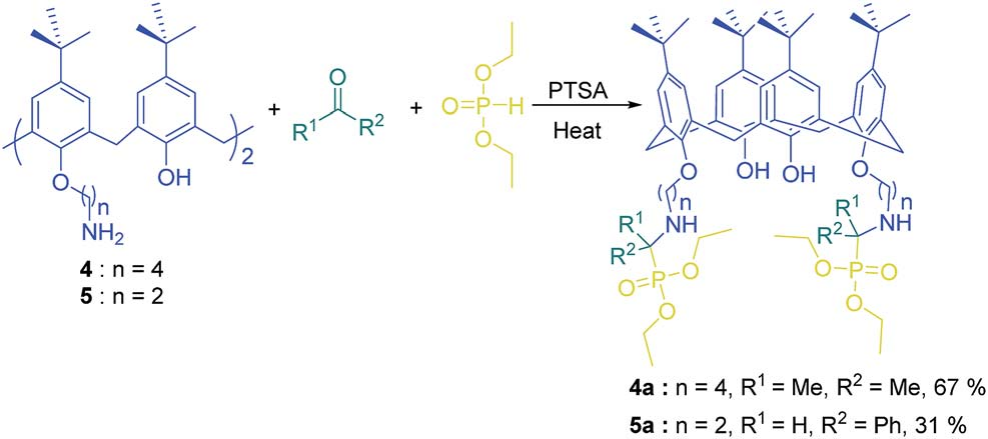
Kabachnik–Fields-3-component reaction of calixarene diamine 4 and 5.

Kabachnik–Fields reaction, a named 3-component reaction of an aldehyde/ketone with an amine and a di-/trialkyl phosphite, provides a useful procedure to yield α-aminophosphonate derivatives.^56,57^ These valuable organophosphorus compounds have many intriguing biological activities. Actually, they are key substrates to obtain α-aminophosphosphoric acids which are α-amino acids analogues and can exhibit a broad range of biological activities. A series of novel calix[4]arene based α-aminophosphonate derivatives were described by Stoikov and his coworkers through Kabachnik–Fields reaction. In this reaction the amino alkyl derivatives of *p-tert*-butylcalix[4]arene (compounds 4 and 5) were used as the platform to construct α-aminophosphonate fragments at the lower rim of the calix[4]arene annulus (Scheme 9).

**Scheme 10:**
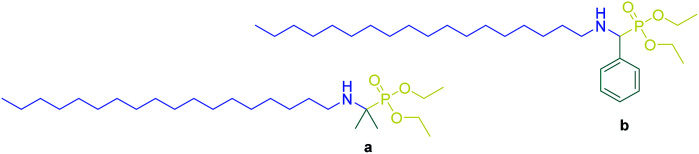
Two acyclic α-amino phosphonates a and b as transmembrane carriers for hydroxycarboxylic acids.

Previously, acyclic α-aminophosphonates such as diethyl(2-(octadecylamino)propan-2-yl)phosphonate a and diethyl((octadecylamino) (phenyl)methyl)phosphonate b were successfully used as an efficient and selective carrier to transport some diacids and α-hydroxy acids through a certain liquid-impregnated membrane (Scheme 10).^58^

But in this work, the use of *p-tert*-butylcalix[4]arene as a platform with three-dimensional structure could modify the selectivity through preorganization of binding sites.

**Fig. 4:**
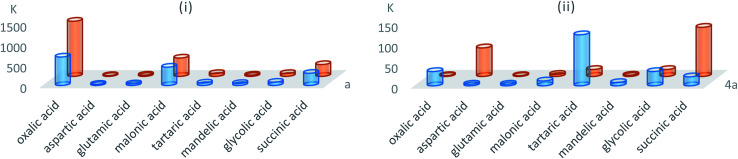
Transport enhancement coefficients (*K* = transfer flux through a liquid-impregnated membrane with carrier/transfer flux through a liquid-impregnated membrane without carrier) for certain organic acids (i) carriers a, b and (ii) carriers 4a, 5a.

The transport ability of compounds 4a and 5a for studied hydroxycarboxylic acids was considerably different from acyclic α-aminophosphonates a and b (Fig. 4). This implies the higher degree of preorganization of functional substituents and the importance of structural and geometric correlations between the binding sites and substrates.^59^

**Scheme 11:**
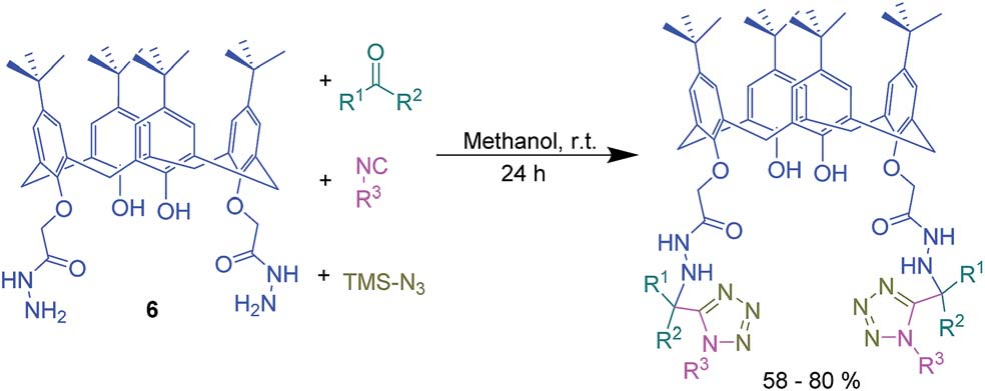
Ugi-azide-4-component reaction of calixarene dihydrazide 6.

In another study, novel lower rim α-hydrazinotetrazolocalix[4]arene derivatives were achieved by Ugi-azide-4-component reaction of calixarene dihydrazide 6 with an aliphatic ketone compound, cyclohexyl or *tert*-butyl isocyanide, and trimethylsilyl azide (Scheme 11). The developed protocol is an operationally simple method to afford the desired products in good yields under mild conditions.

**Scheme 12:**
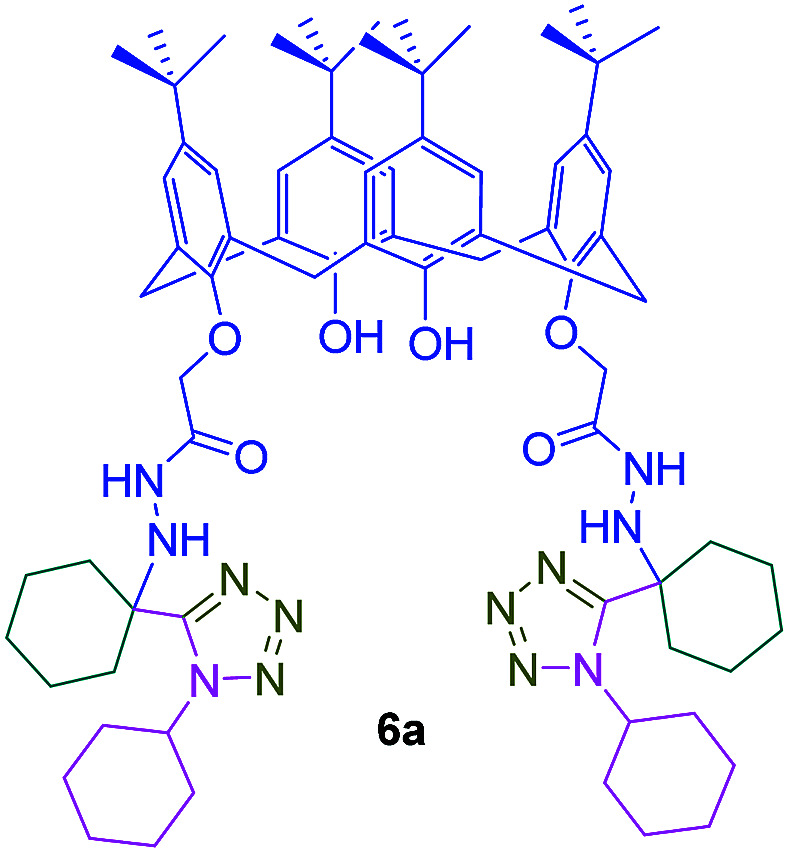
Compound 6a as a selective host for Ni^2+^ cation.

Furthermore, the metal cation binding properties of compound 6a as the model compound were investigated by fluorescence titration with cations as metal perchlorates (Scheme 12).

**Fig. 5:**
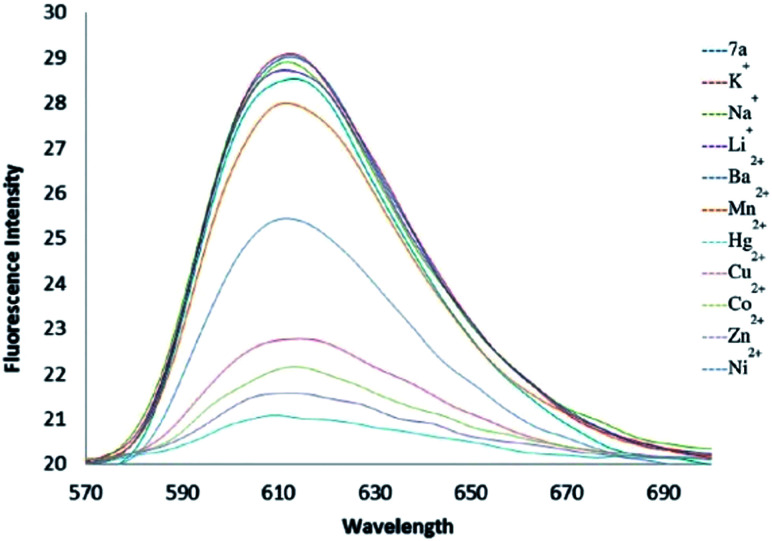
Fluorescence intensity changes of compound 6a in CH_3_CN upon addition of 1 equivalent of various metal perchlorates.

**Fig. 6:**
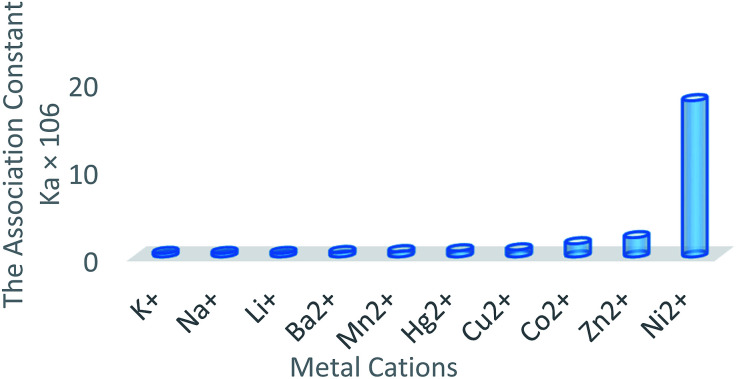
The association constants of 6a with metal cations (1 : 1 binding model) in CH_3_CN.

As shown in Fig. 5, fluorescence quenching study indicated that compound 6a toward Ni^2+^ had the highest association constant (*K*_a_ = 1.70 × 10^7^ M^−1^) to form a Ni : 6a complex. Fluorescent sensors for Ni^2+^ detection are rare and in most of cases, they have serious interference problems from other heavy and transition metal ions. The compound 6a as a specific fluorescent sensor can form complex with Ni^2+^ even in the presence of other metal ions such as Li^+^, Na^+^, K^+^, Ba^2+^, Mn^2+^, Co^2+^, Cu^2+^, Zn^2+^, and Hg^2+^ at 1 equivalent (Fig. 6).^11^

## Upper rim functionalized calix[4]arene derivatives

**Scheme 13:**
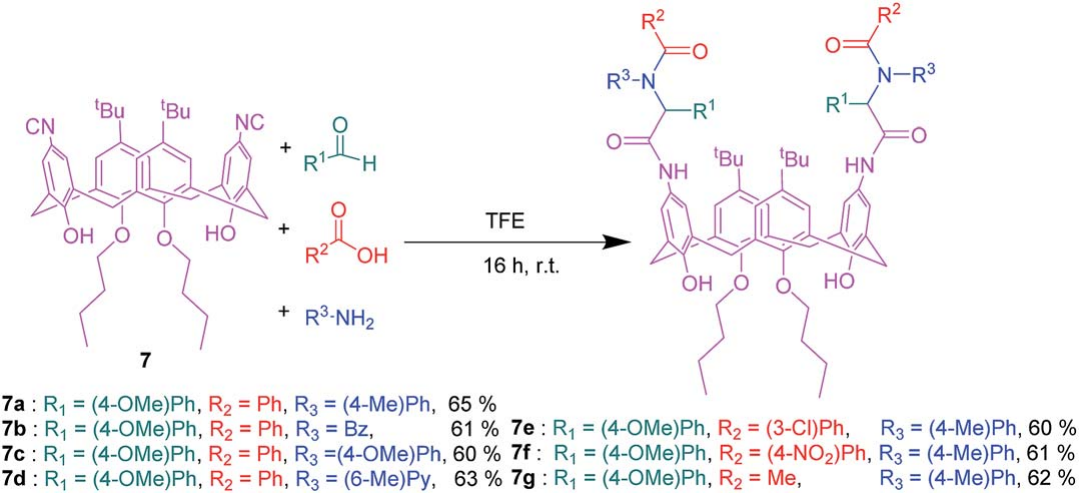
Ugi-4-component reaction of isocyanocalix[4]arene 7.

**Scheme 14:**
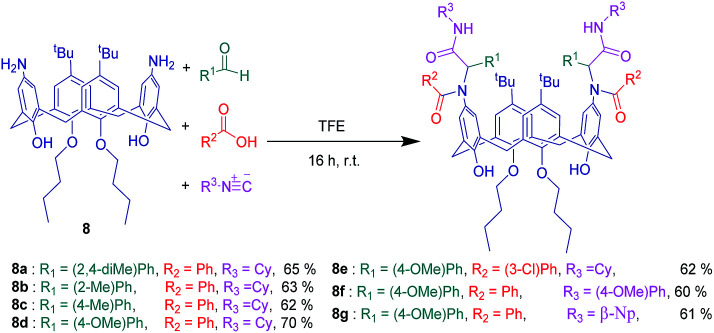
Ugi-4-component reaction of aminocalix[4]arene 8.

Upper rim functionalization is generally more complicated than lower rim. The first step is to remove *tert*-butyl groups from the upper rim that is carried out in good yields by the use of AlCl_3_.^60^ Until now, an enormous amount of effort has been devoted by a large number of researchers to develop diverse functional groups at the upper rim, thereby a fairly extended library of upper rim substituted calixarenes have been available.^61^ Recently, some of upper rim functionalized calix[4]arenes have been used in several MCRs. As the first attempt Varma and coworkers utilized novel isocyanocalix[4]arene 7 and aminocalix[4]arene 8 separately in Ugi-4CR in order to achieve two sorts of calixarene based α-acylaminocarboxamide derivatives (Schemes 13 and 14). This synthetic method provides a general and straightforward route to establish peptoide chains with heteroaromatic and chromophore substitutions on the upper rim. Unlike many other reported peptidocalixarenes, these compounds have excellent solubility in non-polar solvents.^41^

**Scheme 15:**
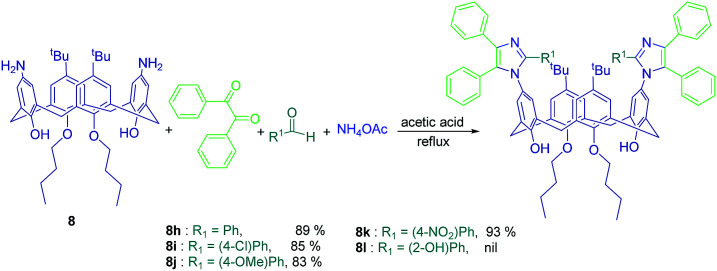
Imidazole-calix[4]arene derivatives synthesis from 4-component reaction of compound 8.

Calix[4]arene diamine 8 was also used by Deng *et al.* in a simple one-pot 4-CR. This reaction was carried out between one equivalent of compound 8 and two equivalent of an aldehyde, benzil, and ammonium acetate in refluxing acetic acid to construct two imidazole rings, which are directly linked to the upper rim (Scheme 15).^62^

**Scheme 16:**
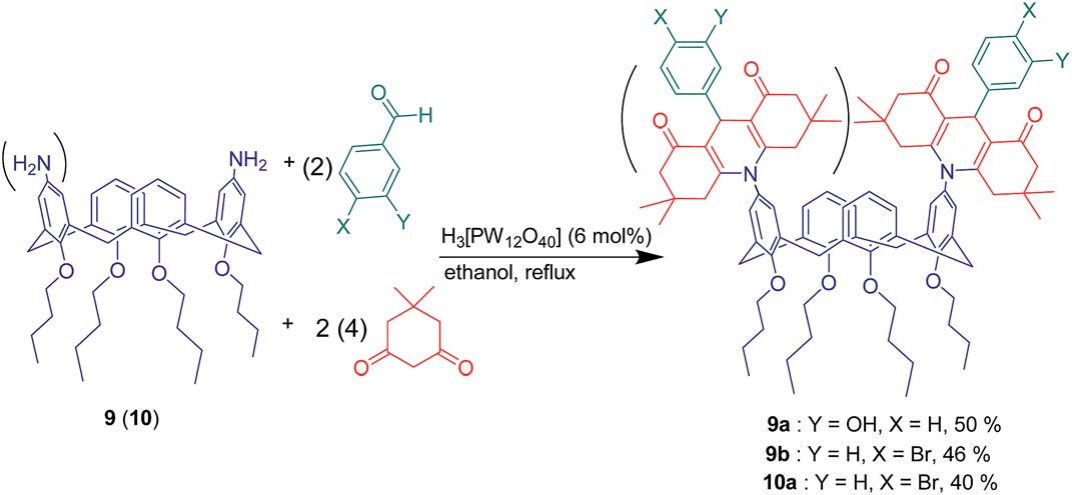
Acridine-calix[4]arene derivatives synthesis from 3-component reaction of compound 9 (10).

Two similar upper rim aminocalix[4]arenes 9 and 10 were applied in a 3-component approach by Mirza-Aghayan *et al.* In order to synthesize three novel acridine-calix[4]arene derivatives 9a, 9b and 10a (Scheme 16). The reaction was conducted in the presence of 6% tungstophosphoric acid hydrate as an efficient heteropolyacid catalyst in boiling ethanol as solvent.

Acridine derivatives have remarkable ability to reversibly intercalate into the helical structure of DNA, so the interaction of acridine-functionalized calix[4]arene derivatives with calf thymus DNA (CT-DNA) was investigated *via* fluorescence titration experiments in aqueous solution. The obtained results showed compound 9b has a larger association constant (*K*_f_ = 2.18 × 10^7^) than compound 9a (*K*_f_ = 3.75 × 10^6^). Furthermore, association constant in compound 10a (*K*_f_ = 6.36 × 10^7^) revealed that the increase of the number of acridine moieties could not considerably improve the affinity toward CT-DNA.^63^

**Scheme 17:**
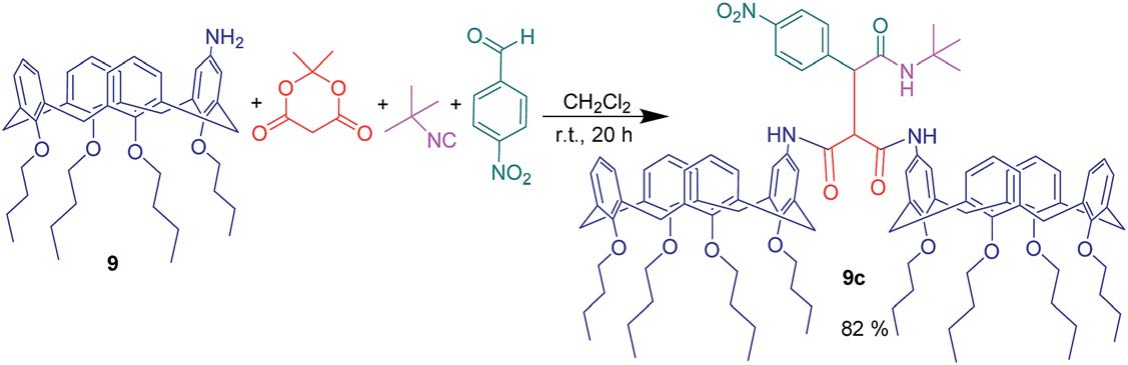
Pseudo-five-component reaction of mono-aminocalix[4]arene 9.

Along the same lines, a highly selective fluorescent chemosensor for NADH was described *via* a pseudo-five-component reaction of two equivalent of mono-aminocalix[4]arene 9 with 4-nitrobenzaldehyde, Meldrum's acid, and *tert*-butyl isocyanide in CH_2_Cl_2_ (Scheme 17).

**Fig. 7:**
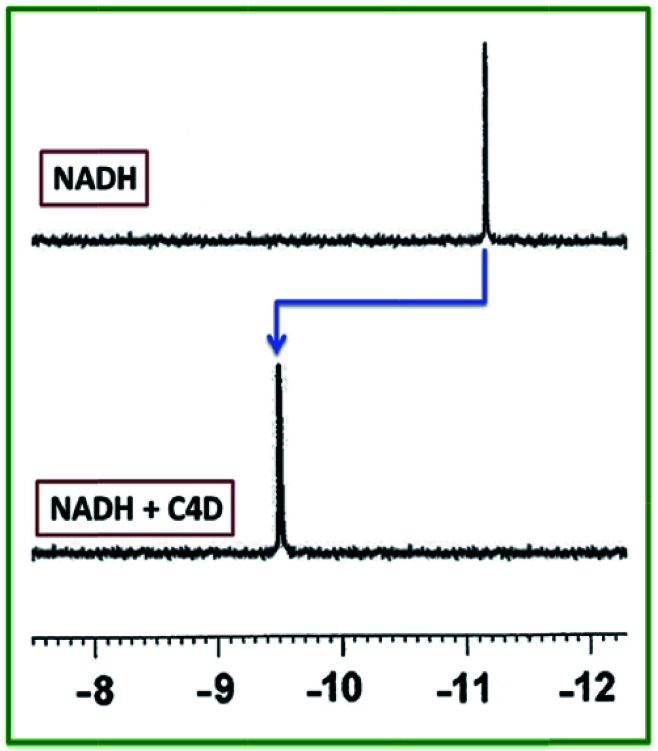
^31^P NMR spectra of NADH and NADH with equivalent amount of 9c in D_2_O.

This reaction led to formation of a dimeric structure of calix[4]arene. The product showed unique selectivity toward NADH to form a 1 : 1 stoichiometric complex and was able to detect at least 16.3 μg L^−1^ NADH over various biomolecules with a binding constant of 3.74 × 10^5^ M^−1^. Actually, for compound 9c, the variation of the fluorescence intensity during complex formation with NADH in the presence of equal amounts of different biomolecules was almost constant. Additionally, the ^31^P NMR spectra of NADH was significantly changed after formation of complex with compound 9c which indicated that NADH phosphate groups interact with the amide moieties of the chemosensor through hydrogen bonding (Fig. 7).^16^

**Scheme 18:**
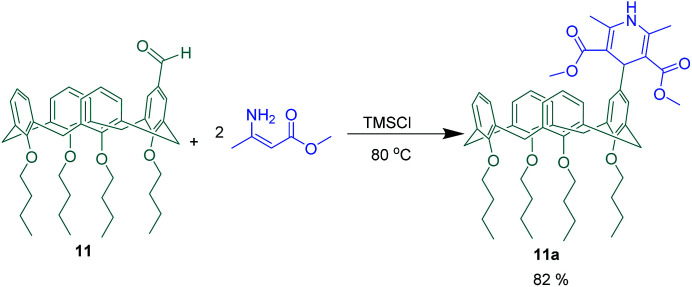
1,4-Dihydropyridine-calix[4]arene synthesis from pseudo 3-component reaction of compound 11.

**Scheme 19:**
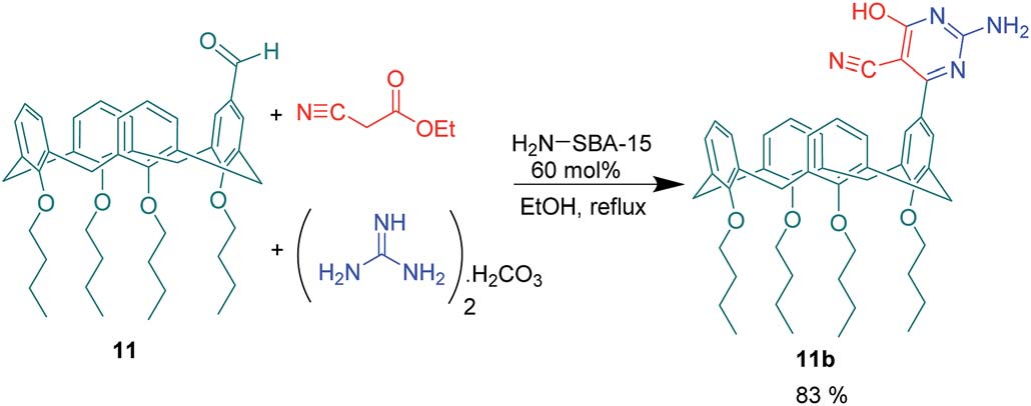
2-Amino-pyrimidine-calix[4]arene synthesis from 3-component reaction of compound 11.

Two new calix[4]arene based receptors 11a and 11b were introduced for saccharide recognition in aqueous solution by Mirza-Aghayan *et al.* For this purpose, the mono-aldehyde calix[4]arene 11 as a carbonyl compound was used in two different MCRs (Schemes 18 and 19). In the first one, novel 1,4-dihydropyridine-calix[4]arene 11a was achieved through a pseudo 3-CR of compound 11 with two equivalent of methyl 3-aminocrotonate in the presence of TMSCl. The interaction of 1,4-dihydropyridine-calix[4]arene 11a toward some mono-and disaccharides was investigated *via* fluorescence and ^1^H NMR titration. The obtained results revealed that compound 11a has a 31-fold greater binding affinity for sorbitol (2.35 × 10^5^ M^−1^) relative to mannitol (7.50 × 10^3^ M^−1^), but lone 1,4-dihydropyridine didn't show any selectivity between the two sugar alcohols. In continuation of this study, 2-amino-pyrimidine-calix[4]arene 11b was afforded through a 3-CR between the mono-aldehyde calix[4]arene 11, ethyl cyanoacetate, guanidinium carbonate and catalytic amount of NH_2_-SBA-15. The product as a saccharide recognizer displayed highest binding affinity toward glucose (3.13 × 10^5^ M^−1^) and fructose (3.60 × 10^5^ M^−1^) over the other saccharides.^64^

**Scheme 20:**
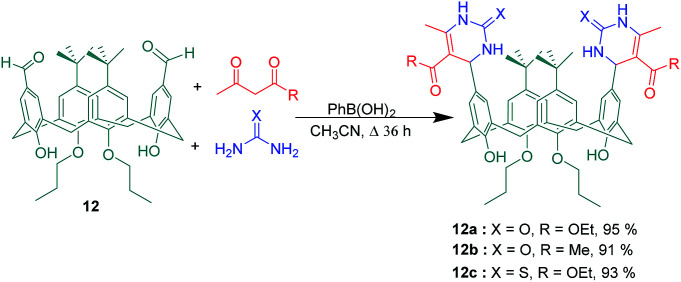
Biginelli-3-component reaction of calixarene dialdehyde 12.

Another use of upper-rim aldehyde substituted calix[4]arene was presented by Varma *et al.* (Scheme 20). In this work, a solution of dialdehyde 12, ethyl acetoacetate, and urea in acetonitrile was heated under reflux in the presence of catalytic amount (10 mol%) phenylboronic acid to afford the corresponding dihydropyrimidine derivative 12a in quantitative yield. One of the special features of this case is direct connection of aromatic nucleus to the stereo centres in two dihydro pyrimidine substituents. In addition, two further upper rim dihydropyrimidine substituted calix[4]arenes 12b and 12c were synthesized through replacement of ethyl acetoacetate by acetyl acetone and urea by thiourea. Although these three calix[4]arene derivatives showed no significant binding affinity toward investigated cations and anions, the study of the aggregation behaviour by ^1^H NMR spectroscopy in CDCl_3_ revealed compound 12a tends to aggregate due to its intermolecular hydrogen bonds in solution.^51^

## Conclusion

In conclusion, the examples highlighted in this review demonstrate that calix[4]arene derivatives can be successfully used to produce highly functionalized building blocks by MCRs in a convenient manner with a minimum of synthetic steps. As previously described, most of the products have outstanding properties in supramolecular chemistry which can be applied for chemical sensing purposes, such as recognition of small ions or even large biological macromolecules, like proteins and nucleic acids. Over the last decade, only four-membered ring calixarenes have been applied in MCRs, so there is potential to develop MCRs by other sizes of calixarene annulus. The diversity of the products obtained by multicomponent reactions in combination with multi-valency properties of calixarenes inspire chemists to develop various highly functionalized derivatives of calixarenes as new multivalent ligands. Additionally, the supramolecular properties of these new products, such as establishment of non-covalent interactions and self-complimentary may be useful to encapsulate certain kinds of drugs or genes to deliver them to specific tissues. It seems that in the coming decade there will be an increase in the number of multicomponent methods for functionalizing calixarene derivatives. Of course, it is expected the final product will be more specialized and more purposeful.

## Conflicts of interest

There are no conflicts to declare.

## Supplementary Material
